# Consistent long-distance foraging flights across years and seasons at colony level in a neotropical bat

**DOI:** 10.1098/rsbl.2024.0424

**Published:** 2024-12-04

**Authors:** María C. Calderón-Capote, Mariëlle L. van Toor, M. Teague O’Mara, Travis D. Bayer, Margaret C. Crofoot, Dina K. N. Dechmann

**Affiliations:** ^1^ Max Planck Institute of Animal Behavior, Am Obstberg 1, Radolfzell 78315, Germany; ^2^ Department of Biology, University of Konstanz, Universitätsstraße 10, Konstanz 78464, Germany; ^3^ Smithsonian Tropical Research Institute, Gamboa, Panama; ^4^ Centre for Ecology and Evolution in Microbial Model Systems, Linnaeus University, Kalmar 391 82, Sweden; ^5^ Bat Conservation International, Austin, TX, USA; ^6^ Department of Biological Sciences, Southeastern Louisiana University, Hammond, LA, USA; ^7^ Cluster for the Advanced Study of Collective Behavior, University of Konstanz, Universitätsstraße 10, Konstanz 78464, Germany

**Keywords:** colony, central-place foraging, exploitation, foraging fidelity, long-distance foraging

## Abstract

All foraging animals face a trade-off: how much time should they invest in exploitation of known resources versus exploration to discover new resources? For group-living central place foragers, this balance is challenging. Due to the nature of their movement patterns, exploration and exploitation are often mutually exclusive, while the availability of social information may discourage individuals from exploring. To examine these trade-offs, we GPS-tracked groups of greater spear-nosed bats (*Phyllostomus hastatus*) from three colonies on Isla Colón, Panamá. During the dry season, when these omnivores forage on the nectar of unpredictable balsa flowers, bats consistently travelled long distances to remote, colony-specific foraging areas, bypassing flowering trees closer to their roosts. They continued using these areas in the wet season, when feeding on a diverse, presumably ubiquitous diet, but also visited other, similarly distant foraging areas. Foraging areas were shared within but not always between colonies. Our longitudinal dataset suggests that bats from each colony invest in long-distance commutes to socially learned shared foraging areas, bypassing other available food patches. Rather than exploring nearby resources, these bats exploit colony-specific foraging locations that appear to be culturally transmitted. These results give insight into how social animals might diverge from optimal foraging.

## 1. Introduction

Foraging is a vital and direct determinant of organismal fitness. Foraging animals have to maintain a delicate balance between exploitation (i.e. directed movements to repetitively use the same patch) and exploration (i.e. tortuous movements due to searching; [1–5]). They must weigh the decision to exploit known but possibly depleting resources against seeking out new, possibly more abundant ones but with added search cost and the risk of being unsuccessful [2,6]. This balance hinges on three main factors: environmental conditions (i.e. quality and quantity of available resources), individual traits (prior information, cognitive abilities/spatiotemporal memory) and social interactions. Social interactions are especially important, as social central place foragers often forage in the presence of others and can learn from them [7–10]. In fact, using social information in changing environments can help balance the decision to exploit versus explore [1,11,12]. Understanding how animals navigate this trade-off is essential for uncovering group dynamics and the development of potential social traditions.

A typical social central place foraging species is the greater spear-nosed bat (*Phyllostomus hastatus*). This species is omnivorous and forages within < 10 km of their roost in Trinidad [13,14]. In the dry season, they forage socially on the nectar and pollen of temporally unpredictable but shareable flowering balsa trees (*Ochroma pyramidale*; [15]), and during the wet season, they feed on fruits (i.e. *Cecropia peltata*) and more insects than in the dry season [15]. In contrast, GPS tracking in Panamá revealed *P. hastatus* flying individually > 25 km to their foraging areas when blooming balsa were particularly scarce [16]. This observed intraspecific variation in foraging distance and social behaviour provides a unique opportunity to explore how foraging strategies are influenced by the resource landscape, particularly how bats navigate the trade-off between exploring new resources and exploiting known ones. We tracked foraging *P. hastatus* over 6 years in three colonies during the dry and wet season in Isla Colón, Panamá. Based on the *P. hastatus* literature [13–15], we predicted that (i) in a regular dry season, bats should forage on balsa in groups and within 10 km of their colony. When feeding on ubiquitously distributed fruit and insects during the wet season, we expected individual exploration behaviour closer to the roost (ii) We predicted colonies would use separate foraging areas at least during the dry season to avoid competition for ephemeral flowering balsa trees. (iii) Finally, we expected switching foraging areas between seasons, reflecting shifts in resource availability and distribution. The results of this study will help understand intraspecific variation in social foraging across years and seasons, and the factors that may lead to deviations from optimal foraging.

## 2. Methods

### (a) Tracking *Phyllostomus hastatus* movements

**Figure 1.**
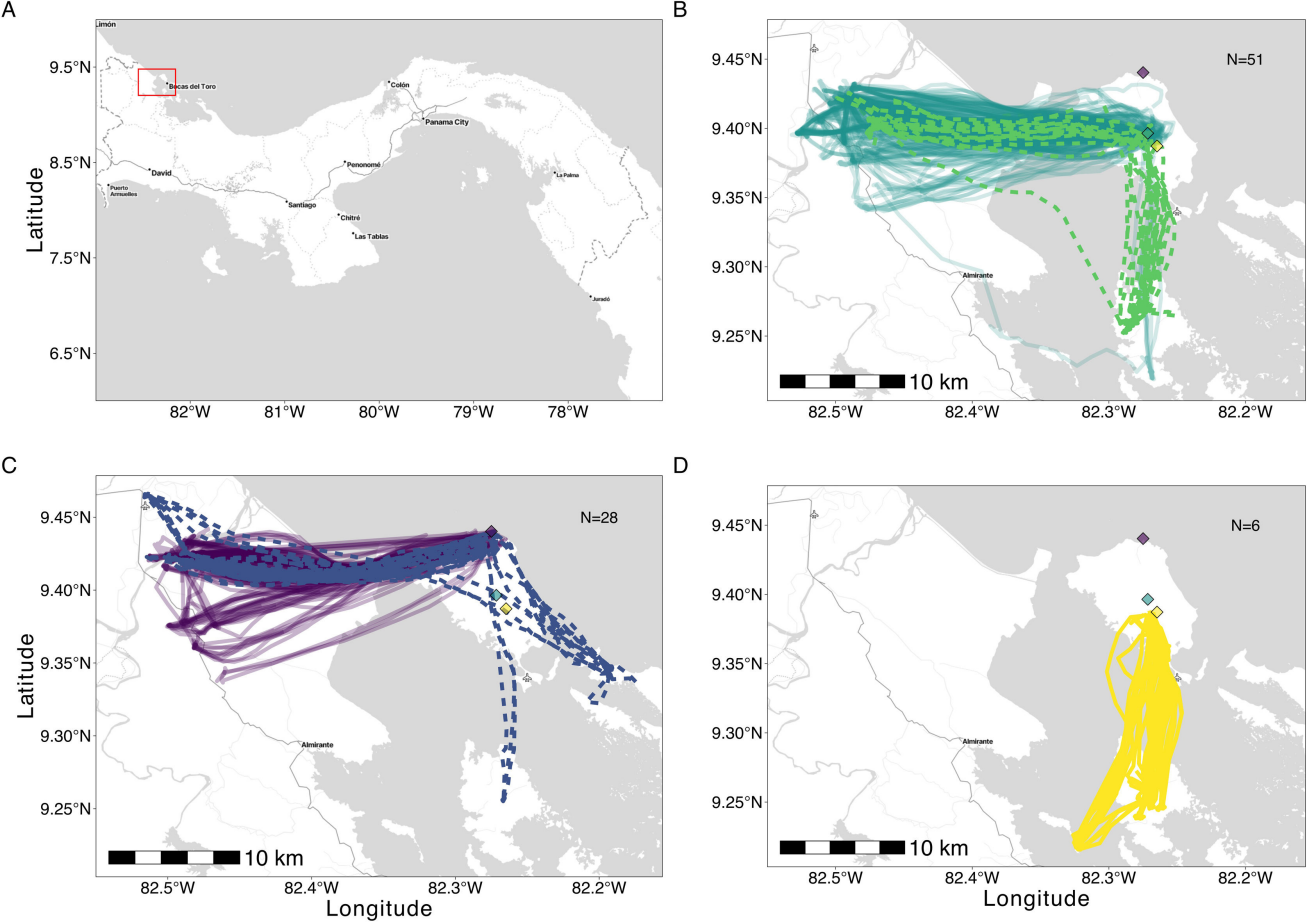
Consistent, colony-specific long-distance foraging flights across years and seasons. (*a*) Map of Panama, inset: study area. (*b*) Colony 1 (wet and dry seasons 2016–2023). (*c*) Colony 2, March 2022 (dry) and August 2023 (wet). (*d*) Colony 3, March 2022 (dry). Roosts (diamonds) colony 1: green, colony 2: purple and yellow: colony 3. Wet season: dotted lines, dry season: solid lines.

We captured 216 individuals (134 females and 82 males) at three different colonies inside caves (details electronic supplementary material, table S1) on Isla Colón, Bocas del Toro, Panamá, during the dry season (February–March) in 2016 [17] and 2022 [18], and wet season (December and August) in 2021 and 2023 ([19]; figure 1).

We used a ring trap to capture bats over roosting cavities. We determined sex, reproductive status and age, measured forearm length (± 0.01 mm), mass (± 0.5 g) and marked them with subcutaneous passive integrated transponders (PIT) tags (ID 100 Transponder, Trovan^®^). We tracked only adults with different biologgers and programming schedules (electronic supplementary material, table S2). Tags were wrapped in shrink tube and glued (Osto-bond, Montreal Ostomy) to bats’ backs. Biologgers weighed 6.18 ± 1.14 g, representing 5.07 ± 1.17 of bat mass (mean ± s.d.). Females were in early pregnancy in March 2022 but did not lose substantial weight after 22–24 days (pre-tagging (*n* = 11): 121.27 ± 8.29 g; post-tagging = 126.81 ± 8.12 g). We recovered a total of 102 tags and used data from 85 individuals for the analysis (electronic supplementary material, tables S2 and S3). Tags collected data from 18.00−06.00 h local time, covering the full period of bats' foraging activity.

## 3. Movement analysis

GPS outliers (fixes recorded over water outside the bats’ foraging range) and points with speeds > 15 m s^−1^ (unlikely for this species) were removed from the data. We downsampled GPS data to 2 min interval (or 3 min interval in March 2022) to correct for different sampling rates (electronic supplementary material, table S2). We used tracking nights with complete outbound and inbound commutes (229 of 420 nights) to calculate foraging time spent on and off the island. From tracks that missed out- or inbound commutes, we only calculated mean distances and directions which were used in the shared foraging distance/angle analysis (simulations).

### (a) Behavioural classification

We fitted a three-state hidden Markov model (HMM) for each bat night using the momentuHMM package to identify behaviours [20]. To implement the HMM, we first regularized the tracks by inserting ‘NA’ for missing observations to obtain a complete series of 2 or 3 min intervals, using the setNA function from the adehabitatLT package [21]. A previous study found social resting between foraging as an important behaviour [16]. However, after downsampling the data resolution we did not accurately distinguish between the categories used there (slow/fast foraging and resting). Thus, we fitted a two-state model with ‘foraging’ (short movements with low persistence of direction including potential resting) and ‘commuting’ (fast and directed movement) as categories even though three-state models had lower AICs. The model was fitted using step lengths (assuming states could be described using a mixture of gamma distributions [20]), and turning angles, with wrapped Cauchy distributions [20]. Behavioural categories were also corroborated by visual inspection after the classification.

### (b) Foraging parameters

We calculated the straightness index (SI) for each outbound commute (*n* = 62, electronic supplementary material, table S4). The SI ranges from 0 (tortuous movements) to 1 (straight movements; details electronic supplementary material, table S4). We extracted foraging points from the previous HMM model for individual complete nights (*n* = 59, electronic supplementary material, table S3) and calculated the proportion of time bats spent foraging on or off Isla Colón (mainland/other islands > 15 km). We tested differences in foraging on and off Isla Colón with a binomial generalized linear model (GLM), first by season, using tracking period and location of foraging as fixed effects, then testing sex differences using tracking period and sex as fixed effects. Significance threshold was *p* ≤ 0.05.

### (c) Simulations and bearings

We simulated alternative tracks reflecting the movement of the tracked bats (details in electronic supplementary material, appendix 1). We aimed to show how the collected data (consistent foraging distance and angle from the colony) deviated from expectations (shorter distances, more exploration and greater commuting angle variation from the colony, especially in the wet season) given the landscape availability.

### (d) Contrasting foraging distance and bearing between colonies and seasons

We determined foraging locations from simulated and observed tracks to compare them. We retained only the first foraging location of each foraging bout with greater than one location. We determined the proportion of foraging locations on and off Isla Colón for simulated and observed foraging locations (electronic supplementary material, figure S2). For each foraging location, we calculated the angle and distance to the colony and compared how means and variances differed between simulation and observation using a multivariate model. This was restricted to foraging locations off Isla Colón as they represented the majority of foraging.

We fitted a linear model of angles and distances to estimate agreement between colonies and seasons for observed foraging locations (equations in electronic supplementary material, table S5) and between simulations and observations. We included multiple observations of individuals as a random effect. We fit the model separately for each colony in the wet and dry seasons for observed and simulated data, including weakly regularizing priors.

We further computed contrasts to evaluate hypotheses. Contrasts—differences between the distributions of parameters estimated by the model—were calculated to determine population mean differences, effective standard deviation and individual-level variability between wet and dry season for each colony. Contrasts were calculated as wet/dry season for angle and distance parameters. We calculated contrasts per colony and season to assess the agreement between observed and simulated foraging locations. We derived a spatial representation of the model estimates to test if similar angles and distances imply shared foraging space between colonies. We estimated the percentage of overlap between colonies during the dry season using the contours of the probability density functions (PDFs), clipped to land only. Models were implemented in STAN via CmdStan (v. 2.34.1) and CmdStanR (v. 0.8.1.9000).

## 4. Results

### (a) Mainland foraging and long-distance commutes in both dry and wet seasons

All bats with at least one completely tracked night (*n* = 59, electronic supplementary material, table S3) predominantly used distant foraging locations, crossing to the mainland or other islands. However, 48 bats also foraged on Isla Colón during both seasons, comprising > 30% of their total foraging (electronic supplementary material, figure S1A, GLM, *p* = 0.01). Females and males spent similar time foraging on and off Isla Colón (electronic supplementary material, figure S1B, *p* (on-island) = 0.25, *p* (off-island) = 0.23). Overall bats from each colony maintained long, straight commutes across seasons (figure 1, electronic supplementary material, table S4).

**Figure 2.**
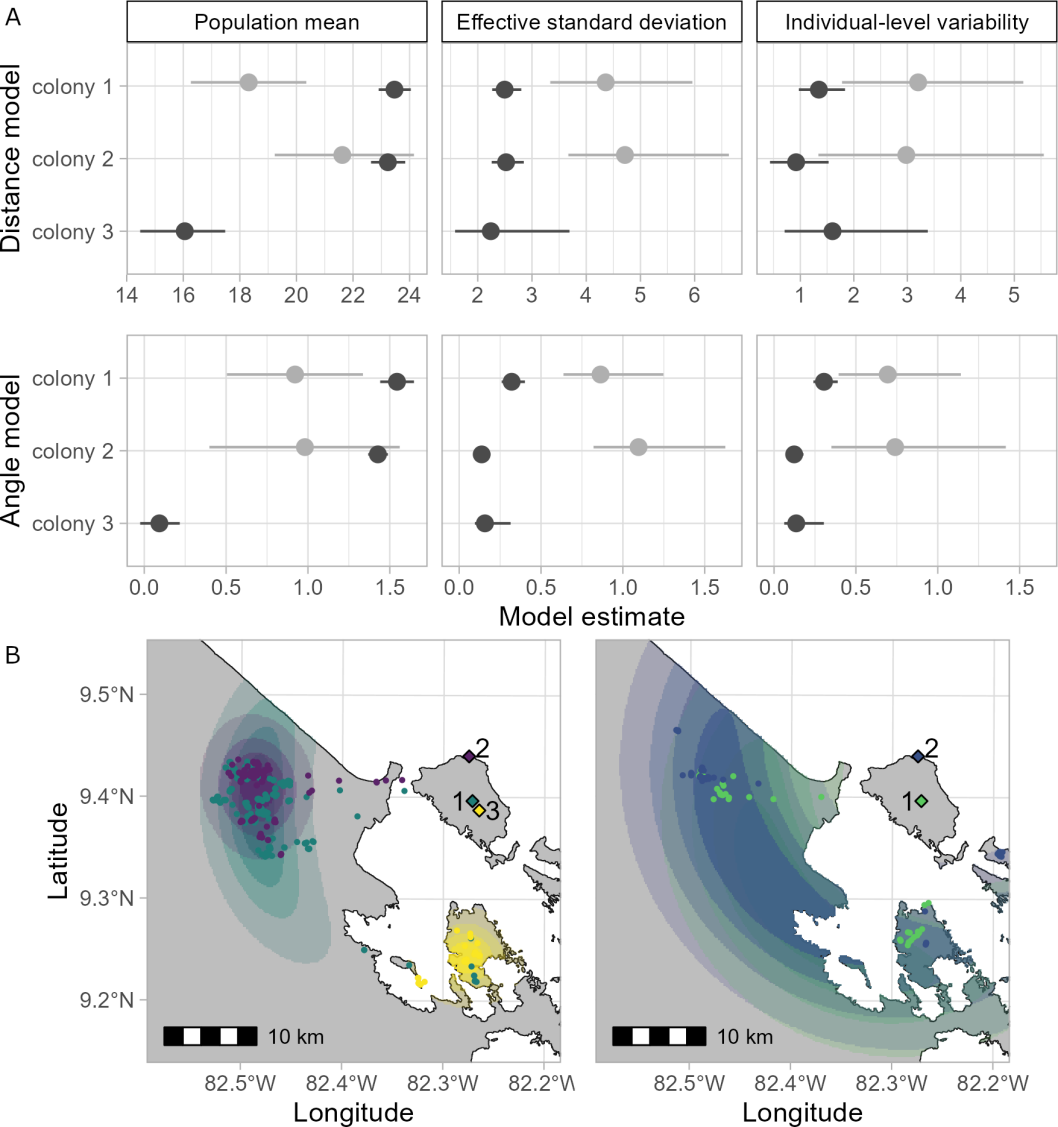
(*a*) Mean and 95% credibility interval for model estimates on population means. Population mean, effective standard deviation and individual-level variability for distance (upper row) and angle from the cave (lower row) to foraging locations. Wet season: light grey, dry season: dark grey. (*b*) Spatial representation of model estimates of foraging locations beyond Isla Colón (dry season; left, wet season: right). Shown are the scaled product of the distance and angle probability density functions, clipped to the 95% contour and coastline. Colony 1: green, colony 2: purple/blue, colony 3: yellow and intensity of colour: relative density of the PDF product.

Bats foraged further from the cave during the dry seasons (mean ranges: 16.05–23.47 km, details table 1, figure 2*a*), with the shortest distance estimated for colony 3 and larger distances for colonies 1 and 2. Mean wet season distance was shorter in colony 1, whereas the model was inconclusive for colony 2.

**Table 1.** Model estimates for population means of distance and angle (estimate and 95% credibility intervals (Qi)) from colonies for observed foraging locations. rad = radians.

colonies	season	mean distance [95% Qi (km)]	mean angle (rad) [95% Qi (rad)]	mean angle (degrees) [95% Qi (degrees)]
colony 1	dry	23.47 [22.91–24.04]	1.54 [1.44–1.65]	268.46 [262.58–274.39]
colony 1	wet	18.31 [16.27–20.35]	0.92 [0.5–1.34]	232.78 [208.93–256.53]
colony 2	dry	23.23 [22.64–23.85]	1.43 [1.37–1.49]	261.77 [258.38–265.29]
colony 2	wet	21.62 [19.23–24.15]	0.98 [0.4–1.56]	236.23 [202.8–269.4]
colony 3	dry	16.05 [14.48–17.48]	0.09 [−0.02–0.22]	185.3 [178.58–192.43]

Distances and angles from the roosting caves varied more in the wet season compared with the dry season (figure 2). Effective standard deviation as well as the deviation of individual means from the population mean were higher during the wet season for both distance and angle (figure 2*a*), albeit varying between colonies and model parameters. Differences were more pronounced for the effective standard deviation than for individual-level variability, with 95% credibility intervals showing a small level of overlap for all but the angle model in colony 2 (figure 2*a*).

### (b) Partial shared foraging distances, directions and space use across seasons and colonies

Individuals within the same colony, and sometimes between colonies shared foraging distances and directions. Foraging areas of colony 1 and 2 were at similar mean angles during both seasons, but colony 3, tracked only during the dry season, foraged at a more southerly site (figure 2*b*, table 1).

The mean estimates suggested that foraging areas of colony 1 and 2 overlapped substantially but colony 3 did not: 49.58% of the area covered by colony 2 overlaps with that of colony 1, and inversely 99.17% of the area covered by colony 1 was shared with colony 2. This was indicated by contours of the PDFs from distance and angle models.

### (c) Assessing observed versus expected space use

Finally, to test our data against our predictions, we compared parameter estimates for observed and simulated foraging locations. The covariates and constraints on the transition probability matrix meant that the model was able to replicate the overall behaviour of the observed trajectories, excluding simulated foraging that fell on the ocean. Mean distance to foraging locations was similar between observations and simulations for colony 1 during the dry season (mean [95% qi]: 21.90 [20.15–23.59] km), but simulated foraging locations were further from the colony for colonies 2 (mean [95% qi]: 29.07 [26.76–31.42] km) and 3 (mean [95% qi]: 37.05 [30.83–43.08] km) than observed locations, respectively. Simulated wet season foraging distances were longer for colony 1 (mean [95% qi]: 21.67 [18.4–24.72] km) but shorter than colony 2 (mean [95% qi]: 16.78 [13.2–20.42] km) than observed (electronic supplementary material, figure S3).

**Figure 3.**
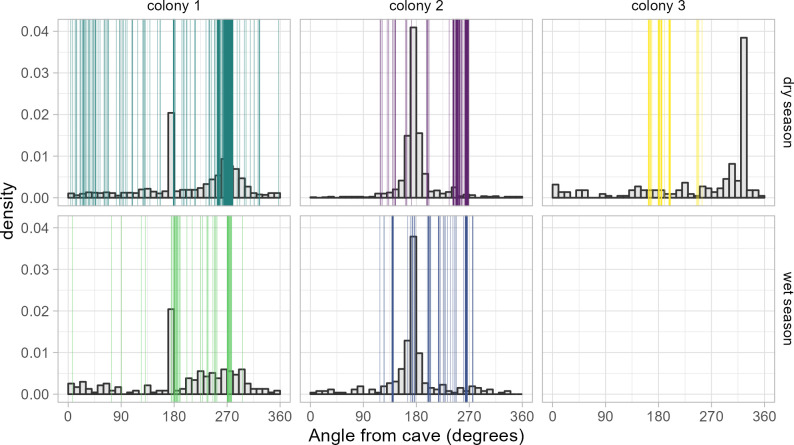
Distribution of angles of outbound commute endpoints from each colony. Histogram: simulated angles of outbound commutes from the colonies based on the available landscape for each colony. Vertical lines (colored): observed angles of endpoints of outbound commutes for individual bats.

When comparing simulated commuting angles with observed ones, the variation was greater for simulated angles, indicating the colonies used less of the available landscape (figure 3). This effect, measured by the effective standard deviation, was notably higher during the dry season for colony 1 (mean [95% qi]: 0.89 [0.82–0.97] rad), colony 2 (mean [95% qi]: 0.69 [0.64–0.74] rad) and colony 3 (mean [95% qi]: 1.17 [0.94–1.47] rad; electronic supplementary material, figure S3). In the wet season, this difference was less pronounced. There was more variation in observed angles of commutes to foraging locations, but still less than in the simulation (effective standard deviation for simulated locations: colony 1: (mean [95% qi]: 0.82 [0.65–1.05] rad), colony 2: (mean [95% qi]: 1.39 [1.19–1.64] rad); electronic supplementary material, figure S3). The simulations were not informed about the distribution of available resources which may have partially influenced the results.

## 5. Discussion

The unique opportunity to follow the foraging behaviour of *P. hastatus* from the same island from 2016 to 2023 revealed consistent colony-level behaviours across years and seasons. Bats consistently used distant foraging sites 15–25 km from the roost (figure 2*b* electronic supplementary material, S4), much further than the < 10 km previously reported [13,14]. Repeated use of distant foraging sites can be beneficial, but the degree and profitability of this behaviour depend on the spatiotemporal predictability, quality and depletability of a given resource [22–24]. The consistent colony-level of foraging areas across years, within seasons, and, with additional foraging areas, between seasons, suggests this behaviour could be due to familiarity [22,25]. Familiarity and the decision to exploit known foraging locations can confer long-term energetic benefits if these locations have higher productivity in temporally unpredictable environments [26]. Consistent foraging patterns help individuals to learn the location of food [27,28], move efficiently through the environment [24,29,30] or reduce conflict with neighbours [31].

Different individuals from different years but from the same colony (colony 1–2) used consistent foraging locations. *Phyllostomus hastatus* is highly social and capable of learning from others [32,33]. It is, thus, possible that this consistency in foraging sites arises through the social transmission of information about the location of profitable resources [34,35], information use at the central place [7,36] or by following others to find unpredictable resources. In Trinidad, this species forms long-term groups of unrelated females that cooperate on multiple levels, including pup-guarding [37] and recruiting others to feeding trees during the dry season [15]. Based on Trinidad’s social system, we expected females to show more similar foraging patterns than males [13], but we observed no difference. The observed long-term foraging fidelity suggests that foraging preferences are learned from others at the colony level rather than from female groups.

We tracked individuals from colony 1 during the early and late dry season of 2022, expecting increased exploration during the latter, i.e. increased path tortuosity/win-stay, lose-shift foraging strategy [38]. With the ongoing season, the switching rate to new foraging areas should match the temporal scale of resource variability (i.e. reduced balsa flower production). Instead, overall site fidelity and path straightness were maintained (figures 1 and 2, electronic supplementary material, table S4), not matching a change in foraging strategy linked to the change in balsa availability over the flowering season and an exploitation instead of exploration strategy. Only one individual exploited a completely different area and another exhibited exploratory behaviour (electronic supplementary material, figure S5A).

The low levels of exploration continued into the wet season. Although foraging distances were shorter in the wet season when feeding on a more ubiquitously distributed diet [15], bats still mostly foraged off Isla Colón (> 15 km). We had also expected less shared foraging space in the wet season. Although some individuals may use more foraging patches in the wet season at the foraging grounds, they continue to use long-distance commutes in a similar direction that we did not expect based on the seasonal shift in diet (figures 1–3, electronic supplementary material, figure S5B). Our results indicate that during part of the year, *P. hastatus* may switch between a set of socially learned foraging areas, rarely exploring individually. Future work will be necessary to investigate how this process works within and across generations of bats.

Long foraging distances in 2016 were thought to be due to unusually late balsa flowering [16]. The continued long foraging distances over the years, when balsa as well as more ubiquitously distributed wet season resources should have been available on the island, were surprising (figures 1 and 2). The use of shared foraging areas is likely a choice rather than a fixed behaviour. Bats spent up to 60–100 min commuting, investing time and effort they could have spent feeding or exploring closer to the roost, avoiding the risk of crossing open water i.e. during strong winds [39]. Why they continue to travel to these distant foraging areas remains unresolved but likely involves learned traditions as they are able to visit and use closer resources (electronic supplementary material, figures S1 and S2).

Individuals from the same colonies consistently used shared foraging distances and direction, though this varied between colonies. We expected this due to competition for limited balsa flowers during the dry season [16]. It is interesting that colony 2 used similar foraging areas to colony 1, even though colony 3 is geographically closer. One possibility is that dispersal of knowledgeable individuals between colonies may have transferred information that spread through the colony. As historical data on these colonies are lacking, we can only speculate about this. Additionally, shared foraging areas could indicate high balsa availability, enough to sustain at least two colonies of 500 bats each [40]. At peak flower production, a balsa tree can feed 3–7 bats over one night [16]. Thus, 72–166 trees would be needed to satisfy the energetic demands of one of these colonies. Ground truthing indicated high balsa availability in these areas, and future studies should incorporate measures of flower availability. Overall, the continued use of a similar area even in the wet season when feeding on insects and fruit reinforces the idea that the use of foraging areas is acquired through memory and possibly conformity, rather than density-dependent factors or between-colony competition, as observed in other frugivorous bats such as *Rousettus aegyptiacus* [28].

Additional non-exclusive aspects may influence the use of shared foraging areas. Bats tracked during 2016 did not share flowering trees but rested together between foraging bouts, potentially to exchange information or increase vigilance against predators [16]. Resolution of tracking data after 2016 was lower to increase the duration of data collection. This made it impossible to test for social resting, but we confirmed that bats returned to similar foraging patches within the shared foraging area night after night.

Our results indicate strong colony foraging preferences that are independent of seasonality and group composition. However, these results represent only a partial picture of the wide range of behavioural strategies that *P. hastatus* might have. Two main limitations remain unresolved: our inability to track bats for long-term periods and our lack of detailed knowledge of *P. hastatus* diet and resource availability for a species that moves tens of kilometres. Our research usually assumes that animal behaviour is always completely adaptive, but our results suggest that animals can choose foraging behaviours that do not follow predictions of ideal foraging and optimizing returns for reasons not yet understood.

## Data Availability

GPS and reference data are available from the Movebank Data Repository [17–19]. The data description and code are available from the Dryad Digital Repository [41]. Supplementary material is available online [42].
